# Complexities of Management of Atypical Ventricular Fibrillation Storm in a Young Patient With TANGO2

**DOI:** 10.1155/2024/9911781

**Published:** 2024-12-04

**Authors:** M. Cecilia Gonzalez Corcia, Catherine Bradshaw, Efstathia Chronopoulou, Jennifer Shortland, Benjamin O'Sullivan, Tim Murphy

**Affiliations:** ^1^Paediatric Cardiology Department, Bristol Royal Hospital for Children, Upper Maudlin Street, Bristol BS2 8BJ, UK; ^2^Paediatric Surgical Department, Bristol Royal Hospital for Children, Upper Maudlin Street, Bristol BS2 8BJ, UK; ^3^Paediatric Metabolic Department, Bristol Royal Hospital for Children, Upper Maudlin Street BS2 8BJ, Bristol, UK; ^4^Paediatric Cardiac Anaesthetic Department, Bristol Royal Hospital for Children, Upper Maudlin Street, Bristol BS2 8BJ, UK

**Keywords:** left cardiac sympathetic denervation, paediatric implantable cardioverter defibrillator, TANGO2, ventricular fibrillation, video-assisted thoracoscopy

## Abstract

TANGO2 deficiency disorder, a rare autosomal recessive genetic disorder characterised by biallelic loss-of-function variants in the TANGO2 gene, was first described in 2016. This disorder involves the transport and Golgi organisation homologue, impacting Golgi membrane redistribution into the endoplasmic reticulum. Clinically, affected individuals exhibit a multiorgan phenotype, with prominent neurological manifestations such as developmental delay and regression. Metabolic crises, triggered by minor infections or fasting, are a hallmark of the disorder. We present the case of a 5-year-old boy diagnosed with TANGO2 deficiency disorder who experienced a refractory-to-treatment ventricular arrhythmia storm. The patient's clinical course was marked by rhabdomyolysis-induced muscle pain, weakness and dark urine. Despite aggressive medical management during intercurrent illness, including hyperhydration, maintenance of normoglycemia and correction of electrolyte abnormalities, the patient's condition deteriorated, leading to a life-threatening ventricular arrhythmia storm. In a life-saving therapeutic approach, the patient underwent a thoracoscopic left sympathectomy during extracorporeal membrane oxygenation (ECMO) support. Remarkably, this intervention resulted in the termination of the ventricular arrhythmia storm. The case underscores the challenges in managing TANGO2 deficiency disorder-associated complications and highlights the potential role of innovative interventions, such as sympathectomy during ECMO, in critical and refractory cases. This case contributes to the understanding of the clinical spectrum of TANGO2 deficiency disorder and emphasises the need for further research into targeted therapies for this rare metabolic condition, where current treatment strategies focus on symptom management.

## 1. Introduction

TANGO2 is a rare genetic disorder first described in 2016 [1]. It has an autosomal recessive inheritance with a biallelic loss-of-function variant in the transport and Golgi organisation 2 homologue (TANGO2) gene [2]. This gene encodes a protein postulated to be involved in redistributing the Golgi membranes into the endoplasmic reticulum (ER), and as such, it is categorised under the metabolic conditions, with multiorgan phenotype. Affected individuals present with neurological impairment manifesting as developmental delay and regression [3]. Moreover, they experience metabolic crises triggered by trivial infections or fasting. These episodes typically start with rhabdomyolysis resulting in muscle pain, weakness and dark urine but have a potential for encephalopathy and lethal arrhythmias. In addition, there can be abnormalities in the 12-lead electrocardiogram (ECG) and a risk of ventricular arrhythmias. At present, there is no cure for the disorder, and therapies are aimed at management of the specific symptoms. The mainstay of medical management is during intercurrent illness and consists of hyperhydration aimed at the preservation of renal function during rhabdomyolysis, maintaining normoglycaemia, aggressive management of electrolyte abnormalities, especially magnesium, and cardiac monitoring in the intensive care setting for the prompt medical treatment of arrhythmias. We present the case of a 5-year-old boy with a TANGO2 deficiency disorder with a refractory-to-treatment ventricular arrhythmia storm that terminated during a thoracoscopic left sympathectomy during extracorporeal membrane oxygenation (ECMO) support.

### 1.1. Methods

Informed consent was obtained from the affected individual's guardians, including consent to publish photographs. Clinical information was retrieved both from paper and electronic medical records.

## 2. Case Report

### 2.1. Initial Presentation and Diagnosis

A 12-month-old boy, with a background of developmental regression, including inability to crawl, inability to stand when supported and limited ability to sit without support, was presented with a first episode of rhabdomyolysis including muscle pain, weakness and dark urine. Of note, there was no personal or family history of seizure disorders or epilepsy, nor was there any personal history of growth retardation, with the patient maintaining normal percentiles in the growth charts. Laboratory tests at this time revealed a creatine phosphokinase (CPK) level above 400,000, which is markedly elevated. Of interest, the acylcarnitine profile and urine organic acid tests were within normal values, which excluded a metabolic crisis. The symptoms appeared shortly after his immunisations. An echocardiogram performed during this period was normal, but his ECG showed a prolonged QTc of 533 milliseconds. A muscle biopsy was conducted, revealing nonspecific muscle fibre necrosis, and electron microscopy was nondiagnostic. A whole-exome sequencing was performed on genomic DNA from blood sample. The genetic testing was performed at the National Health System (NHS) Highly Specialised Service for Rare Mitochondrial Disorders at Newcastle University. The test consisted of an automated sequencing of the TANGO2 gene to screen for reported or potential pathogenic mutations and long-range PCR to screen for reported or potential pathogenic mutations and long-range PCR to screen for the 34kb founder deletion [1]. The methodology involved amplification of the coding the region of the TANGO2 gene using M13-tagged intronic primers and screening by direct sequencing of the PCR-amplified products and multiplex long-range PCR to screen for a large-scale TANGO2 rearrangement. Data analysis was performed using the Mutation Surveyor software (V5.0.1) and Alamut software (V2.10). The nomenclature used adheres to Human Genome Variation Society (HGVS) guidelines, based on sequence accession number NM_152906.6 [4]. The test revealed evidence of a homozygous large-scale deletion of around 35kb involving TANGO2 in the patient's DNA sample. No other inherited arrhythmia disorders could be identified in a 64-gene arrhythmia panel. Furthermore, parental genetic tests confirmed both parents were carriers of the gene mutation for TANGO2.

**Figure 1:**
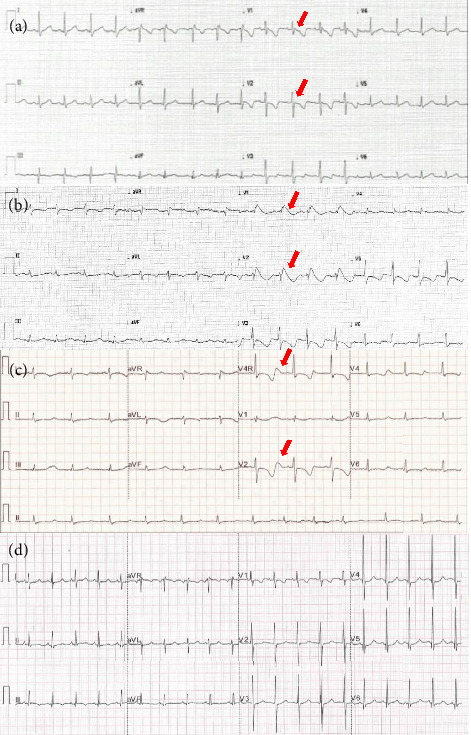
12-lead electrocardiogram. (a): Normal baseline ECG out metabolic crises. Note: the small r′ in V1 (red arrows) but normal J point position. The QT interval is 341 ms with a corrected QT interval of 400 ms. (b): 12-Lead electrocardiogram at admission with normally placed electrodes (no high-lead positioning). Note: PR interval of 160 ms, QRS prolongation with fragmentation and J point elevation in the precordial leads V1 and V2 (red arrows), QT interval prolongation with corrected QT at 500 ms and T-wave inversion in the left leads and all precordial leads. (c): Last presympathectomy ECG performed while still in sinus rhythm. Note: the very prolonged QT/QTc intervals in the order of 580/630 ms respectively. The arrows point to intermittent presence of T-wave alternance. (d): ECG the day after sympathectomy. The QT interval is 320 ms with a corrected QT interval of 435 ms, and no J point elevation (red arrows).

Following recovery, he was managed with avoidance of fasting, an MCT-enriched diet, and supplements of coenzyme Q10 (200 mg once daily), riboflavin (50 mg twice a day), and magnesium glycerophosphate (194 mg three times a day) with a weight at admission of 16.5 kilos. During intercurrent illness, he was admitted for hyperhydration and ECG monitoring (Figure 1(a) shows normal baseline ECG), with emphasis on maintaining magnesium at the upper limit of normal. It is noteworthy that intercrisis ECG consistently showed normal QT/QTc intervals within the range of 420–450 ms. At the age of 4 years, he had another major episode of rhabdomyolysis with QT prolongation, with very similar treatment and outcomes. He evolved with developmental delay but was able to walk and started to speak single words, with some degree of dysarthria.

### 2.2. Acute Onset of Arrhythmic Event

At the age of 5 years, the patient presented with symptoms of afebrile general malaise, feeling tired but with no signs of viral infection, two days after receiving the first dose of COVID-19 vaccination. There had been no change in his chronic medications, no missed doses, no vomiting or dietary alteration. Initial laboratory tests demonstrated normal CPK levels of 15 U/L (normal reference range for age according to NHS United Kingdom laboratories < 190 U/L), normal liver function tests including aspartate transaminase and alanine transaminase (AST 22 U/L and 24 U/L, respectively, within the normal range for the lab), and normal glycaemia and electrolytes. Subsequently, serial daily CPK, liver tests and electrolytes remained within normal limits (range from 33 to 77 U/L). Twelve-lead ECGs, including high precordial leads, were performed at admission and every 12–24 h, meticulously monitoring intervals including PR, QRS length and QT.

The initial 12-lead ECG showed a type I Brugada pattern (Figure 1(b)), which reverted with the initial fluid and electrolyte therapy and did not recur, although QT/QTc intervals remained at ranges of 500–550 ms. Due to this high-risk type 1 ECG, he was transferred from the peripheral hospital to our centre on the second day of admission. Repeated ECG showed a very prolonged QT/QTc interval in the order of 580/630 ms, respectively, with intermittent presence of T-wave alternance (Figure 1(c)). Echocardiograms were performed on admission and on daily or every-other-day basis to follow on heart function. Muscle enzymes were also monitored with daily blood tests and remained normal.

Initial acute treatment included fluid, glucose and electrolyte replacement, with target magnesium level > 2.2 mg/dL. Oral nutrition was enrichened with medium-chain triglyceride formula (Liquigen®) 30 mL three times a day, along with coenzyme Q10 (200 mg oral daily), riboflavin (50 mg twice a day) and magnesium glycerophosphate (dose 10mmol = 242 mg of magnesium three times a day). Electrolytes were corrected on a regular basis according to the laboratory results.

Total parenteral nutrition (TPN, Bespoke®) was initiated on the third of admission, consisting of a once-daily reconstitution formula (Solivito N®) containing vitamin B1 (thiamine mononitrate, dose 3.1 mg), vitamin B2 (riboflavin, dose 4.9 mg), vitamin B3 (niacin/nicotinamide, dose 40 mg), vitamin 5 (sodium pantothenate, dose 16.5 mg), vitamin B6 (pyridoxine/dose 4.9 mg), vitamin B7 (biotin dose 60 micrograms), vitamin B9 (folic acid, dose 400 micrograms), vitamin B12 (cyanocobalamin, dose 5 micrograms), amino acid glycine (dose 300 mg) and vitamin C (sodium ascorbate 113 mg). Additionally, this TPN formula was complemented by a high-potency formula (Pabrinex®) containing vitamin B1 (thiamine, dose 125 mg/day), B2 (riboflavin, dose 2 mg/day), B3 (niacin/nicotinamide, dose 80 mg/day), B6 (pyridoxine, dose 25 mg/day) and vitamin C (ascorbic acid, dose 250 mg/day). Thyroid hormone and cortisol levels were closely monitored, and prophylactic cortisol replacement (iv hydrocortisone) was initiated on the fourth day of admission.

**Figure 2:**
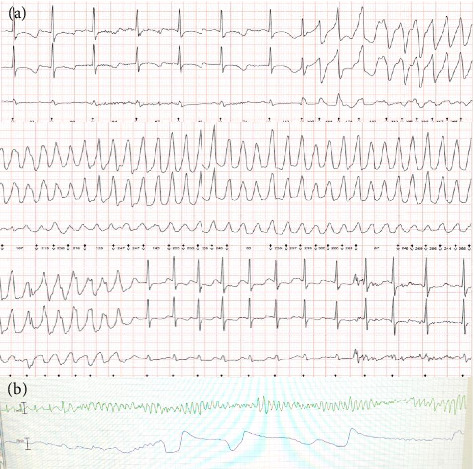
Ventricular arrhythmia presentation. (a): 3-Lead Holter tracing showing onset and termination of nonsustained polymorphic ventricular arrhythmia lasting 10 s, exhibiting features resembling Torsades de Pointes. Note: the onset with a ventricular triplet during the period of relative bradycardia (heart rate around 70 beats per minute). (b): Monitor photography of episode of persistent ventricular fibrillation while on ECMO.

Episodes of nonsustained ventricular fibrillation (VF) started on the day of admission before any treatment and became more frequent despite oral beta-blockers (propranolol 2 mg/kg/day in 3 doses).  Figure 2 depicts the onset of VF during a period of relative sinus bradycardia (heart rate 70 bpm). Propranolol was discontinued, and an intravenous infusion of isoproterenol 0.05 mcg/kg was started in the PICU, where he was intubated and ventilated. Over the next 48 h, euglycaemia and electrolyte homeostasis were strictly targeted, and the isoproterenol infusion was titrated up to 1 mcg/kg/min to achieve heart rates of 130–140 beats per minute, resulting in transient abolition of the arrhythmias. However, during the next 36 h, the patient presented a progressive breakthrough of VF requiring multiple isoproterenol boluses and direct current cardioversions (DCCVs). Transvenous pacing was considered, but the patient presented with incessant VF on arrival in the catheterisation laboratory and required ECMO support as an emergency procedure.

**Figure 3:**
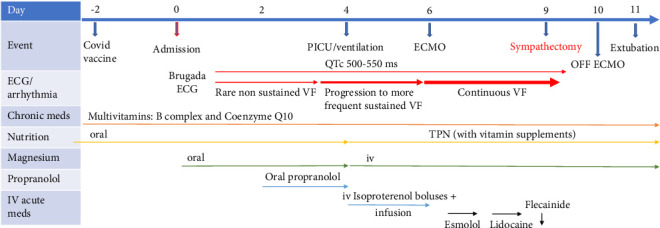
Timeline in days from onset of symptoms to extubation and discharge from PICU.

During the next 3 days, continuous VF persisted on ECMO. Several intravenous antiarrhythmic strategies failed to control the arrhythmia storm: intravenous boluses of isoproterenol and magnesium proved ineffective; an esmolol bolus followed by an infusion was then attempted for a period of 6 hours. Subsequently, intravenous lidocaine (initial dose: 1 mg/kg iv, followed by an infusion started at 20 mcg/kg/min and titrated up to 50 mcg/kg/min) failed as well. Finally, a single dose of intravenous flecainide (2 mg/kg over 10 min) was administered, again unsuccessfully. The intravenous antiarrhythmic drugs we used generally have a short half-life (with the exception of flecainide): isoproterenol 5 min, esmolol 9 min and lidocaine 1 h. Even if there could be some unavoidable drug overlap, appropriate precautions were taken to minimise concurrency and assure a protected time for single drug action for each new choice of medication (Figure 3). It is noteworthy that the QTc interval remained prolonged (ranging from 480 to 550 ms) throughout the whole medical management period.

Of note, serial echocardiograms during sinus rhythm consistently demonstrated normal ejection fraction. He was referred to a transplant centre but was not viewed as a suitable heart transplant candidate due to his underlying metabolic condition. The option of a video-assisted thoracoscopic sympathectomy under ECMO, as a life-saving procedure, was discussed and agreed upon with a multidisciplinary team.

Throughout his admission, a team consisting of specialists in cardiology, electrophysiology, intensive care, metabolic medicine, endocrinology, nutrition, thoracic surgery and cardiac anaesthesia contributed to the care and held twice-daily meetings to discuss management. Advice was also received from world experts.

### 2.3. Thoracoscopic Left Sympathectomy—Surgical Technique

A video-assisted thoracoscopic left sympathectomy was performed under general anaesthesia, following established protocols outlined in previous adult and paediatric literature [5–7]. The patient was on ECMO support, which added an extra layer of complexity to the procedure. The standard protocol for anticoagulation on ECMO, consisting of a continuous intravenous heparin infusion, was continued during the procedure.

Initially, the patient was placed in a modified right lateral decubitus position (50 degrees) with left shoulder and elbow in flexion. The baseline rhythm at the beginning of the procedure was sinus rhythm alternating with sustained and nonsustained periods of VF. Access to the pleural cavity was obtained by three small incisions using 5 mm balloon ports. A 5-mm/30-degree thoracoscope (Stryker Endoscopy, Santa Clara, California) was inserted through the fifth intercostal space anterolateral to the tip of the scapula. Two additional ports were triangulated in the fourth and sixth intercostal spaces at the level of the anterior axillary line.

Preservation of the pleura during previous open chest ECMO cannulation facilitated the creation of a pneumothorax, which was necessary to compress the left lung (despite minimal ventilation on ECMO), in order to obtain adequate views of the left thoracic sympathetic chain. The sympathetic chain was identified running vertically in the costovertebral region under the parietal pleura and isolated. The parietal pleura was dissected using a hook diathermy to expose the left sympathetic chain from ribs 1 to 5. With care to preserve intercostal vascular bundles, the left sympathetic chain was excised as a single specimen with a hook diathermy from the fifth intercostal space to the level of the subclavian vein at the thoracic inlet. Tension permitted the stellate ganglion to be delivered below this level for the excision with scissors on diathermy. Minimal haemostasis was required. No chest tube was placed at the end of the operation, and the left lung was allowed to completely re-expand.

By the end of the procedure, there was no evidence of ventricular arrhythmia, and the patient left the operating room in sinus rhythm. The procedure did not present any complications, including bleeding under anticoagulation.

### 2.4. Postsympathectomy Outcome

Following the sympathectomy procedure, the patient remained in sinus rhythm with the absence of any further ventricular arrhythmia, and with a normal 12-lead ECG. Interestingly, the previous ECG, which allowed QTc measurement (when the patient was in sinus rhythm), was dated 3 days before and showed a prolonged QTc interval in the range of 480–550 ms. Figure 1(d) depicts the normalisation of the ECG following the procedure, with completely normal ST segment, T-wave and QT/QTc intervals. The ECMO support was successfully weaned and discontinued, with evidence of normal cardiac function while on normal sinus rhythm.

On day 2 postprocedure, an epicardial single chamber implantable cardioverter defibrillator was implanted, and the chest was closed. The patient was subsequently extubated and showed complete neurological recovery to his baseline developmental status. He was transferred to his local hospital on no antiarrhythmic therapy on the 10th day postprocedure.

It is noted that the patient displayed a left-sided Horner's syndrome, as would be expected after a left-sided sympathectomy, including partial excision of the stellate ganglion.

### 2.5. Perioperative General Anaesthetic Management

The patient underwent three procedures under general anaesthesia, and the anaesthetic management was carefully discussed amongst members of the multidisciplinary team. While guidelines for managing patients with long QTc syndromes exist, principally the avoidance of drugs known to lengthen the QTc interval (with propofol appearing to be safe, though debate persists about the safety of the volatile agents), the extreme rarity of the TANGO2 mutation complicates decision-making regarding anesthetic management. Similarly, recognised guidelines for the anaesthetic management of patients with mitochondrial disorders suggest avoiding propofol infusions, although single boluses appear to be safe and well-tolerated.

## 3. Discussion

This is a case report of a unique case of life-threatening sustained ventricular arrhythmias in a young patient with the TANGO2 variant. Several aspects of the management strategy employed are highlighted.

First, the clinical presentation is noteworthy for its uniqueness: the patient experienced multiple episodes of rhabdomyolysis without abnormal ECGs or arrhythmias between 12 months and 5 years of age, followed by a markedly abnormal ECG that progressed to a VF storm without associated rhabdomyolysis. The failure to diagnose a febrile or metabolic abnormality, despite serial daily assessments, renders this case unusual, as the presentation cannot be classified as a “metabolic crisis.” Additionally, the episodes of ventricular arrhythmias were not related to electrolyte derangements, rhabdomyolysis did not occur, and electrolyte levels typically remained within the normal range during arrhythmias. Furthermore, systolic function during periods of sinus rhythm was preserved throughout the episode, prompting a very timely wean of ECMO once the arrhythmia storm was under control. Surprisingly, the patient was discharged home with normal 12-lead ECG and cardiac function 7 days after the sympathectomy was performed.

Regarding the pharmacotherapy implemented during the hospitalisation, initial treatment with beta-blockers did not help to shorten the QTc interval or to prevent the onset of progressive ventricular arrhythmias. As a second-line strategy, a chronotropic agent, isoproterenol, was elected. As its beta-1 agonist effect increases heart rate, isoproterenol is sometimes used to manage bradycardia-induced ventricular arrhythmias in inherited cardiac conditions including long QT syndrome and Brugada syndrome. While the initial boluses of isoproterenol succeeded in terminating the arrhythmia, they proved insufficient to stabilise the rhythm in the longer term, even if an appropriate tachycardia was achieved. The transient effect of the bolus could not be maintained by the intravenous infusion even at high rates. Subsequent trials with an intravenous beta-blocker (esmolol), lidocaine, and the single dose of flecainide were administered as last-resort therapies once the patient was supported by ECMO, in an attempt to terminate the now continuous VF.

The challenging development of refractory VF, despite multiple antiarrhythmic therapies in a patient supported on ECMO, prompted a decision to request an urgent evaluation for transplant at the national centre. However, the patient was excluded as a candidate given his underlying metabolic condition. It is acknowledged that transplant policies and legislations vary within countries and geographic areas, and the TANGO2 mutation may not preclude transplant in some regions of the world [8].

A thoracoscopic sympathectomy approach to perform left cardiac sympathetic denervation in a child supported on ECMO has previously been reported [9]. This case contributes to the limited existing literature by presenting a second patient who experienced acute termination of arrhythmia following a life-saving intervention with a minimally invasive approach and without complications. The efficacy of this procedure lies in the fact that increased sympathetic tone is often related to refractory ventricular tachyarrhythmias, and surgical partial excision of the left stellate ganglion, which provides sympathetic innervation to the heart, has proved effective in treating such ventricular arrhythmias [10].

Regarding this rare inherited condition, recent research has advanced our understanding by reporting on a cohort of young patients presenting with cardiac crisis, including cardiac arrhythmias and cardiomyopathy during TANGO2 deficiency-related metabolic crises. In this series, ventricular arrhythmias were present in 80%, with a lethal outcome in a third. Temporary percutaneous sympathetic block with lidocaine was transiently effective in one patient, although surgical left cardiac sympathetic denervation achieved no positive effect in two patients.

Finally, it is noteworthy that following the sympathectomy, the QT/QTc intervals normalised completely. After a follow-up period of 6 months, the patient did not experience recurrent arrhythmias, type I ECG pattern or prolonged QTc interval in the follow-up ECGs.

## 4. Conclusions

TANGO2 deficiency disorders may present as ventricular fibrillation storms requiring multiple treatment approaches including high dose of vitamins and supplements, multiple antiarrhythmics and innovative approaches including sympathetic denervation.

## Data Availability

The data supporting the findings of this study are available from the corresponding author upon reasonable request. Due to patient privacy and confidentiality concerns, some data may be restricted to protect the patient's identity. Any data sharing will comply with institutional guidelines and ethical approvals.

## References

[1] Kremer L. S., Distelmaier F., Alhaddad B. (2016). Bi-Allelic Truncating Mutations in TANGO2 Cause Infancy-Onset Recurrent Metabolic Crises With Encephalocardiomyopathy. *The American Journal of Human Genetics*.

[2] Lalani S. R., Liu P., Rosenfeld J. A (2016). Recurrent Muscle Weakness With Rhabdomyolysis, Metabolic Crises, and Cardiac Arrhythmia Due to Bi-Allelic TANGO2 Mutations. *The American Journal of Human Genetics*.

[3] (2023). Human Genome Variation Society. https://www.hgvs.org/content/guidelines.

[4] Schwartz P. J., Priori S. G., Cerrone M (2004). Left Cardiac Sympathetic Denervation in the Management of High-Risk Patients Affected by the Long-QT Syndrome. *Circulation*.

[5] Li J., Liu Y., Yang F (2008). Video-Assisted Thoracoscopic Left Cardiac Sympathetic Denervation: A Reliable Minimally Invasive Approach for Congenital Long-QT Syndrome. *The Annals of Thoracic Surgery*.

[6] Atallah J., Fynn-Thompson F., Cecchin F., DiBardino D. J., Walsh E. P., Berul C. I. (2008). Video-Assisted Thoracoscopic Cardiac Denervation: A Potential Novel Therapeutic Option for Children With Intractable Ventricular Arrhythmias. *The Annals of Thoracic Surgery*.

[7] Meisner J. K., Ames E. G., Ahmad A (2020). Heart Transplantation for *TANGO2*-Related Metabolic Encephalopathy and Arrhythmia Syndrome-Associated Cardiomyopathy. *Circulation: Genomic and Precision Medicine*.

[8] Franklin A. D., Llobet J. R., Sobey C. M., Daniels J. M., Kannankeril P. J. (2019). Stellate Ganglion Catheter Effective for Treatment of Ventricular Tachycardia Storm in a Pediatric Patient on Extracorporeal Membrane Oxygenation: A Case Report. *A& Practice*.

[9] Pearce B. (2022). Poster It Takes More Than Two to Tango. *Presented at the ASM of Congenital Cardiac Anesthesia Society’*.

[10] Miyake C. Y., Lay E. J., Beach C. M (2022). Cardiac Crises: Cardiac Arrhythmias and Cardiomyopathy During TANGO2 Deficiency Related Metabolic Crises. *Heart Rhythm*.

